# Supplementary material for: Genomic Features and Molecular Function of a Novel Stress-Tolerant Bacillus halotolerans Strain Isolated from an Extreme Environment
Source: Biology (Basel). 2021 Oct 12;10(10):1030. doi: 10.3390/biology10101030 (PMC8533444; doi:10.3390/biology10101030)
Supplement: Supplementary file 1 [file biology-10-01030-s001.zip › Supplementary Figures S1-S4.pdf]

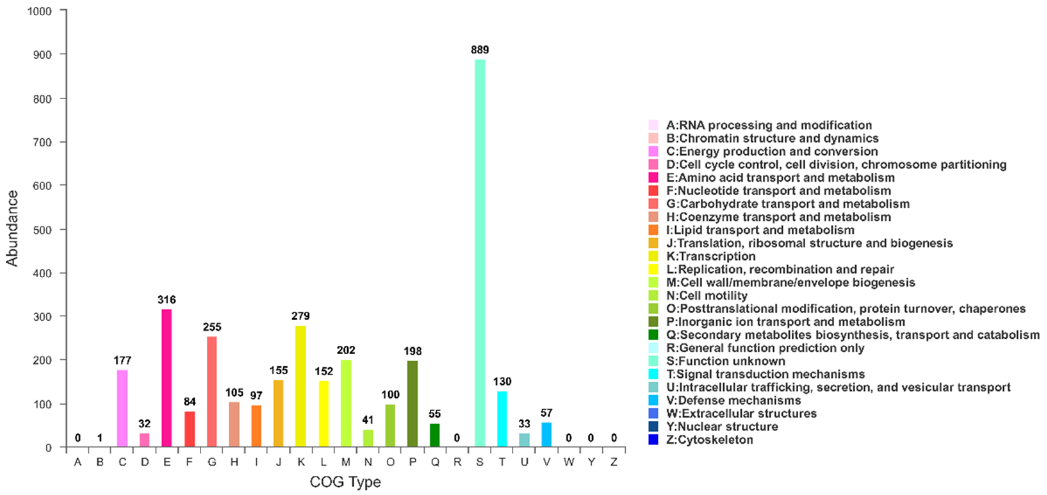

Supplementary Figure S1. Genomic analysis based on COG database.

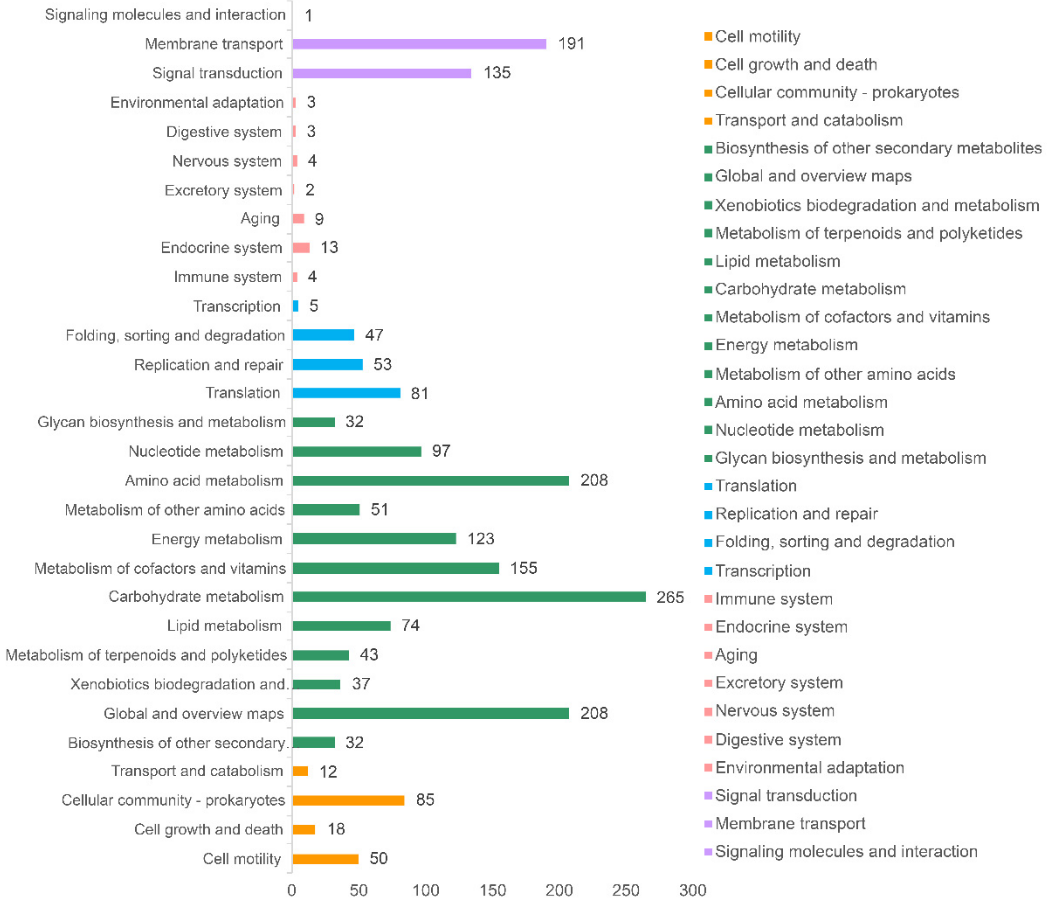

Supplementary Figure S2 Genomic analysis based on KEGG database.

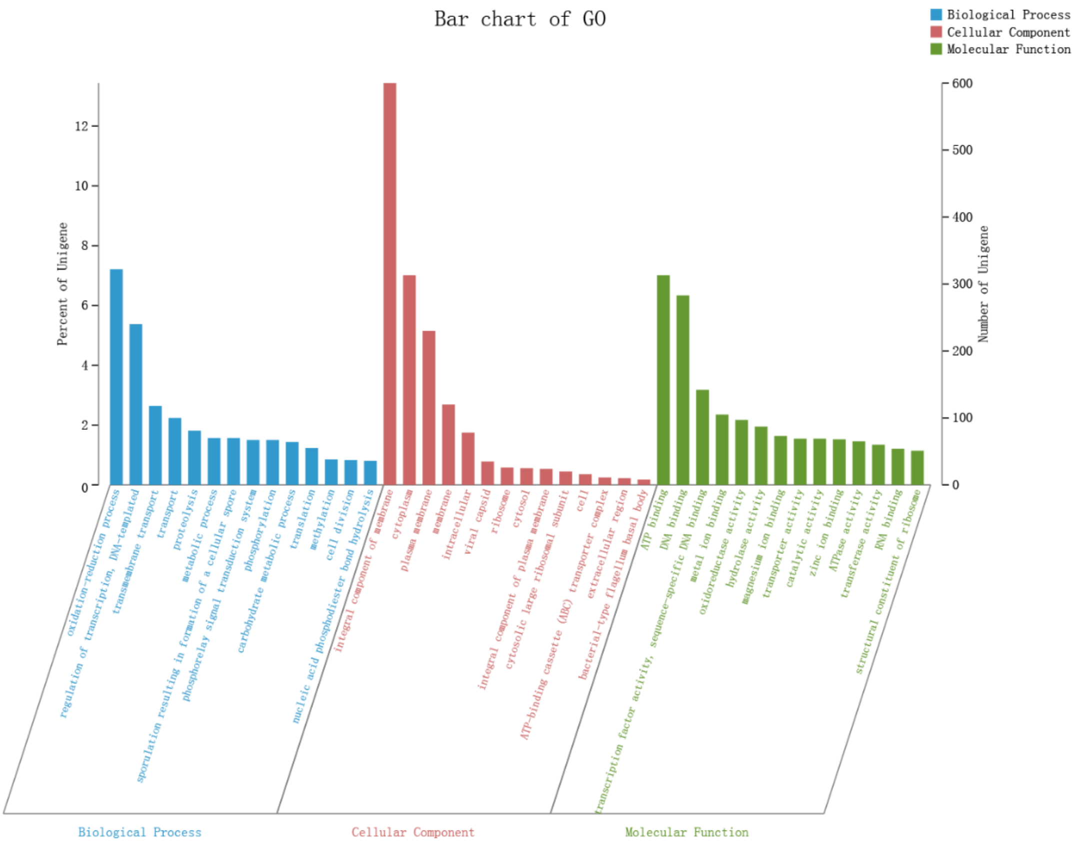

Supplementary Figure S3. Genomic analysis based on GO database.

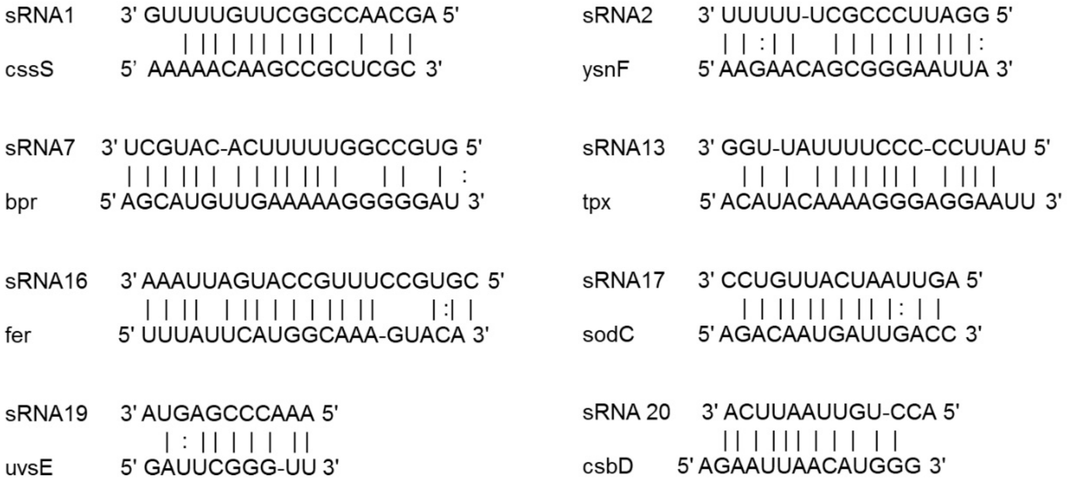

Supplementary Figure S4. The sRNAs and their target mRNA binding sites in *B. halotolerans* KKD1.

Note: The target gene of sRNA1 is *cssS* (encoded sensor histidine kinase CssS), which involved in the basic stimulus - response coupling mechanism. The target gene of sRNA2 is *ysnF* (putative stress response protein), and the target gene of sRNA20 is *csbD* (encoded stress response protein CsbD), which involved in the basic stimulus - response coupling mechanism. These two sRNAs may contribute to

stress-resist in *B. subtilis* KKD1. The target gene of sRNA7 is *bpr*, that encoded for peptidase S8, the are key proteins involved in essential plant physiological processes. The target gene of sRNA13 is *tpx* (encoded 2-Cys peroxiredoxin), which involved in the oxidation-reduction process. The target gene of sRNA17 is *sodC* (encoded superoxide dismutase), protects cells to against various oxidative stress. The target gene of sRNA16 is *fer* (encoded ferredoxins), the small iron-sulfur proteins that participate in various metabolic pathways due to its essential role in plant hormone biosynthesis. The target gene of sRNA19 is *uvsE* (encoded for UV damage endonuclease UvsE), which may assist *B. subtilis* KKD1 to endow the intense ultraviolet irradiation.
