# Supplementary material for: Genomic Features and Molecular Function of a Novel Stress-Tolerant Bacillus halotolerans Strain Isolated from an Extreme Environment
Source: Biology (Basel). 2021 Oct 12;10(10):1030. doi: 10.3390/biology10101030 (PMC8533444; doi:10.3390/biology10101030)
Supplement: Supplementary file 1 [file biology-10-01030-s001.zip › Supplementary Tables S1-S5.pdf]

Supplementary Table S1

Isolation sites of the *Bacillus* strains collected from the QTP.

NO	Strain	Isolation site	Isolation altitude (m)	Isolation temp range (°C)	Host plant	Organ	Soil type	Species according to phylogenomics
1	KKD1	Qinghai, Yushu State, Hoh Xil	4680	-10.0 - 4.1	*Androsace umbellate*	root	Saline-alkali soil	*B. halotolerans*
2	CDHWZ7	Qinghai, Haixi State, Chaidan Lake	2800	-3.0 – 11.0	*Peganum harmala*	root	Saline-alkali soil	*B. velezensis*
3	NLHLT2	Qinghai, Haixi State, Nalanhe country	3010	-3.0 – 13.0	*Tamarix ramosissima*		Desert	*B. atrophaeus*
4	NLHLT8	Qinghai, Haixi State, Nalanhe country	3010	-3.0 – 13.0	*Tamarix ramosissima*	root	Desert	*B. atrophaeus*
5	DGLBC7	Qinghai, Haixi State, Golmud City, Dagele country	2800	0 – 12.0	*Nitraria tangutorum*	root	Desert	*B. atrophaeus*
6	CDHWZD6	Qinghai, Haixi State, Chaidan Lake	2800	-3.0 – 11.0	*Peganum harmala*	root	Saline-alkali soil	*B. atrophaeus*
7	CDHWZS3	Qinghai, Haixi State, Chaidan Lake	2800	-3.0 – 11.0	*Tamarix ramosissima*	root	Saline-alkali soil	*B. atrophaeus*
8	DGLBC2	Qinghai, Haixi State, Golmud City, Dagele country	2800	0 – 12.0	*Nitraria tangutorum*	root	Desert	*B. atrophaeus*
9	CDHWZ2	Qinghai, Haixi State, Chaidan Lake	2800	-3.0 – 11.0	*Suaeda glauca*	root	Saline-alkali soil	*B. atrophaeus*
10	CDHWZS2	Qinghai, Haixi State, Chaidan Lake	2800	-3.0 – 11.0	*Suaeda glauca*	root	Saline-alkali soil	*B. atrophaeus*
11	DGLBC1	Qinghai, Haixi State, Golmud City, Dagele country	2800	0 – 12.0	*Nitraria tangutorum*	root	Desert	*B. atrophaeus*
12	CDHF2	Qinghai, Haixi State, Chaidan Lake	2800	-3.0 – 11.0	*Suaeda glauca*	root	Saline-alkali soil	*B. atrophaeus*
13	HYB6	Qinghai, Xining city, Huangyuan County	2470	-2.0 -12.0	*Brassica napus*	root	Farmland	*B. atrophaeus*
14	HYB7	Qinghai, Xining city, Huangyuan County	2470	-2.0 -12.0	*Brassica napus*	root	Farmland	*B. atrophaeus*
15	HYB8	Qinghai, Xining city, Huangyuan	2470	-2.0 -12.0	*Brassica napus*	root	Farmland	*B. atrophaeus*

		County						
16	HYDDT1	Qinghai, Xining city, Huangyuan County	2470	-2.0 -12.0	*Solanum tuberosum*	root	Farmland	*B. halotolerans*
17	HYDDT3	Qinghai, Xining city, Huangyuan County	2470	-2.0 -12.0	*Solanum tuberosum*	root	Farmland	*B. atrophaeus*
18	HYDDT5	Qinghai, Xining city, Huangyuan County	2470	-2.0 -12.0	*Solanum tuberosum*	root	Farmland	*B. atrophaeus*
19	BHC1	Qinghai, Haixi State, Atomic City	3010	-2.0 -10.0	*Kobresia pygmaea*	root	Grassland	*B. thuringiensis*
20	TSH5	Qinghai, Haixi State, Golmud City,	2980	-2.0 – 11.0	*Suaeda glauca*	root	Saline-alkali soil	*B. atrophaeus*
21	KKLW	Qinghai, Haixi State, Keke Saline Lake	3010	-14 – 12.5	*Phragmites communis*	root	Saline-alkali soil	*B. velezensis*
22	TGRG7	Qinghai, Haixi State, Delingha City	2980	-2.0 – 11.0	*Phragmites communis*	root	Saline-alkali soil	*B. atrophaeus*
23	CDHWZD7	Qinghai, Haixi State, Chaidan Lake	2800	-3.0 – 11.0	*Suaeda glauca*	root	Saline-alkali soil	*B. atrophaeus*
24	TJCD3	Qinghai, Haixi State	2800	0 – 12.0	*Peganum harmala*	root	Desert	*B. atrophaeus*
25	TJCD6	Qinghai, Haixi State	2800	0 – 12.0	*Peganum harmala*	root	Desert	*B. thuringiensis*

Supplementary Table S2

The RT-qPCR primers for detection of genes in this study.

NO.	Oligo Name	Sequence 5' to 3'	Function
1	rspJ-F	GAAACGGCAAAACGTTCTGG	house keeping
	rspJ-R	GTGTTGGGTTCACAATGTCG	
2	proW-F	CTGCTTTCGTATATCTTTTGCCG	salt stress
	proW-R	CGCTTCAGTTGCTTCAATCAG	
3	proV-F	CAGAATACGGACTGGAGCTTC	salt stress
	proV-R	CAAGTTTAAGCGACTCAAGCG	
4	putP-F	TGTATAAGTCGATGTTCCGGC	salt stress
	putP-R	CTCCCAAGCCAGAACTAACG	
5	opuE-F	TGTCCGTCTTAGTGATTGCTG	salt stress
	opuE-R	TTCATCCGCTTCCAATACAGG	
6	proX-F	GGAGACAAAATCAACCTTGCC	salt stress
	proX-R	AGTTTCACGTCATAGCCCAAG	
7	lldE-F	CATTCTCTGTCAAAATGGCGC	salt stress
	lldE-R	TTGTCCTTACGATCAAGCCG	
8	sufB-F	TGACGCTTTCTATCGCTCTTG	salt stress
	sufB-R	ACTTTCCCGCCTTGTTTAGAG	
9	sufD-F	GGAAGACCATAGCTCAGTAACG	salt stress
	sufD-R	GAAGACAGAGTATCCACCGC	
10	bcrA-F	ACAGGAGTTTATTTCCATCCGG	salt stress
	bcrA-R	GAACATCAGCTCTTCAATCGTTG	
11	PRODH 2-F	ATAAGATGGCGAGAAACTGGG	salt stress
	PRODH 2-R	AGTAACTGACAAGCCTTGGC	
12	gene2258-F	CGCAGGTTGATATTACACAATGG	salt stress
	gene2258-R	GATATGTTGCCGCTCCAAGA	
13	genea0525-F	TGATGGTGATATTGACGGCTG	salt stress
	genea0525-R	GATATGTTGCCGCTCCAAGA	
14	treR2-F	AAGGCTCCGTGATTCTCAAC	salt stress
	treR2-R	GGCTGTTTTCGTTTCTTTTCCC	
15	opuD-F	GTTCTCCATTTTCGGTGTTTCC	salt stress
	opuD-R	ATCAATGCCAGGATAGACGTG	
16	opuA-F	CCTAAGCTACTGAAATGGCCG	salt stress

	opuA-F	CCCGTAATACACCTATTCGCTG	
17	treB-F	ACCTTCGCTACAGATTTCCG	salt stress
	treB-R	CATAATCGAGAAAATGCCGGG	
18	nhaC-F	GCTTAATTCCTTATGGCGCG	salt stress
	nhaC-R	CTTGTGAATTTTGGCAGTCCG	
19	glnT-F	GTGCGATTTTCTGGATGTGG	salt stress
	glnT-R	TTGAGCCAGTGTACTTTCGAC	
20	betB-F	TCACGAAGAGACAGAAAGCG	salt stress
	betB-R	CCGTTTTGAAGTGCTGGATC	
21	degU-F	TCGTGAAGGTGTTAAGCGG	salt stress
	degU-R	ATGATAGTGCTCAACGATACGAG	
22	urtD-F	AACCGATACTTATCTGCCGTG	alkali stress
	urtD-R	CTGTACACTGATATCTGCCCC	
23	mnhA-F	ACAAAAGACAGTATCGCCTCC	alkali stress
	mnhA-R	TGTATCAAAGCCCCTGAAGTC	
24	mnhB-F	TCCTCTTCCATCGTTTTGCTG	alkali stress
	mnhB-R	CCGAAGACAAAAGAGCCAAC	
25	mnhC-F	CATCCTTCATCCTCGTCATGG	alkali stress
	mnhC-R	TTCCCCTCATTTGATCCATATCG	
26	mnhD-F	CTGTCGGCGTTATTTTGTTCG	alkali stress
	mnhD-R	GCCTTTAATCAGCATGTCGTG	
27	mnhE-F	AGAGCTGACAAAGGATTGGG	alkali stress
	mnhE-R	TTCCGCATCCTCAATATCCATC	
28	mnhF-F	CGTTAATCCTTCAAATCGCGC	alkali stress
	mnhF-R	GGAATAGTCGGCCCTTTGATG	
29	mnhG-F	TGATTTTGCTCGGTGTCTTC	alkali stress
	mnhG-R	TGATTTTGCTCGGTGTCTTC	
30	nhaC-F	TTATTCCCGTTCTGACTGCC	alkali stress
	nhaC-R	CAGCCTCATTGTCAGTTTCG	
31	yuiF-F	TTATTCCTGTGCTGATACCGC	alkali stress
	yuiF-R	ATATAAGGAGCCGTTAAGCCG	
32	tuaH-F	TTTCGCTTCGGACGGTATAC	alkali stress
	tuaH-R	CAAGGATACAGGGAAGACAGC	

33	clsA-F	ATGTCGGAGGGTTTAATGTCG	alkali stress
	clsA-R	AGGATGAATCGTGTCTGAATGG	
34	tuaG-F	GTCCTGTGCGTTTTCCTTTAC	alkali stress
	tuaG-R	ACCGTTAGACAGCCGATAATG	

Supplementary Table S3

The PCR primers for detection of genes in this study.

NO	Oligo Name	Sequence 5’ to 3’
1	*gyrB*-F	GAAGTCATCATGACCGTTCTGCAYGCNGGNGGNAARTTYGA
	gyrB-R	AGCAGGGTACGGATGTGCGAGCCRTCNACRTCNGCRTCNGTCAT

Supplementary Table S4

NCBI accession numbers of the gyrB and 16SrRNA partial sequences of the QTP isolates

No	Species	strain	16S rRNA accession	length (bp)	gyrB accession	length (bp)
1	*Bacillus halotolerans*	KKD1	CP054584.1		CP054584.1	
2	*Bacillus velezensis*	CDHWZ7	MT912931.1	1,349	MT984373.1	1,056
3	*Bacillus atrophaeus*	NLHLT2			MT984370.1	1,077
4	*Bacillus atrophaeus*	NLHLT8	MT912945.1	1,350	MT984372.1	1,068
5	*Bacillus atrophaeus*	DGLBC7			MT984378.1	1 073
6	*Bacillus atrophaeus*	CDHWZD6	MT912932.1	1,352	MT984374.1	1,071
7	*Bacillus atrophaeus*	CDHWZS3			MT984375.1	1,069
8	*Bacillus atrophaeus*	DGLBC2	MT912923.1	871	MT984371.1	903
9	*Bacillus atrophaeus*	CDHWZ2	MT912930.1	1,349	MT984361.1	1,079
10	*Bacillus atrophaeus*	CDHWZS2	MT912933.1	1,350	MT984376.1	1,079
11	*Bacillus atrophaeus*	DGLBC1			MT984369.1	1,062
12	*Bacillus atrophaeus*	CDHF2	MT912926.1	894	MT984360.1	1,061
13	*Bacillus atrophaeus*	HYB6	MT912937.1	1,348	MT984382.1	1,085
14	*Bacillus atrophaeus*	HYB7	MT912938.1	1,350	MT984380.1	1,082
15	*Bacillus atrophaeus*	HYB8	MT912939.1	1,349	MT984381.1	1,073
16	*Bacillus halotolerans*	HYDDT1	MT912921.1	869	MT984363.1	1,082
17	*Bacillus atrophaeus*	HYDDT3	MT912940.1	1,349	MT984365.1	1,081
18	*Bacillus atrophaeus*	HYDDT5	MT912941.1	1,345	MT984383.1	1,068
19	*Bacillus thuringiensis*	BHC1	MT912927.1	1,358	MT984366.1	1,065
20	*Bacillus atrophaeus*	TSH5	MN849882.1	1,419	MT984368.1	1,075
21	*Bacillus velezensis*	KKLW	CP054714.1		CP054714.1	
22	*Bacillus atrophaeus*	TGRG7	MT912947.1	1,353	MT984377.1	885
23	*Bacillus atrophaeus*	CDHWZD7			MT984364.1	1,070
24	*Bacillus atrophaeus*	TJCD3			MT984379.1	1,068
25	*Bacillus thuringiensis*	TJCD6			MT984367.1	1,069

Supplementary Table S5

Biosynthetic gene clusters occurring in KKD1 and KKLW. Gene clusters occurring in KKD1 are labelled in red. Gene clusters occurring in KKLW are labelled in blue.

Metabolite	MIBiG	Type	Strain	accession	Region	from	to	Most similar known cluster		Similarity
Surfactin	BGC0000433.1	NRPS	KKD1	CP054584.1	1	356,907	420,815	surfactin	NRP-Lipopeptide	86%
Surfactin	BGC0000433.1	NRPS	KKLW	CP054714.1	1	318,362	383,221	surfactin	NRP-Lipopeptide	91%
Fengycin	BGC0001095.1	NRPS	KKD1	CP054584.1	5	1,985,368	2,066,567	fengycin/plipastatin	NRP	100%
Fengycin	BGC0001095.1	NRPS	KKLW	CP054714.1	7	1,850,304	1,987,741	fengycin	NRP	100%
Bacillomycin	BGC0001090.1	NRPS, transAT-PKS	KKLW	CP054714.1	7	1,850,304	1,987,741	bacillomycin D	NRP, Polyketide	100%
Bacillibactin	BGC0000309.1	NRPS	KKD1	CP054584.1	8	3,257,728	3,307,466	bacillibactin	NRP	100%
Bacillibactin	BGC0001185.1	NRPS	KKLW	CP054714.1	12	3,001,616	3,053,404	bacillibactin	NRP	100%
Bacilysin	BGC0000888.1	other	KKD1	CP054584.1	10	3,863,986	3,905,404	bacilysin	other	100%
Bacilysin	BGC0001184.1	other	KKLW	CP054714.1	13	3,579,530	3,620,948	bacilysin	other	100%
Bacillaene	BGC0001089.1	transAT-PKS	KKD1	CP054584.1	4	1,809,061	1,924,069	bacillaene	Polyketide + NRP	100%
Bacillaene	BGC0001089.1	transAT-PKS	KKLW	CP054714.1	6	1,680,571	1,790,182	bacillaene	Polyketide + NRP	100%
Macrolactin	BGC0000181.1	transAT-PKS	KKLW	CP054714.1	5	1,368,802	1,456,945	macrolactin H	Polyketide	100%
Difficidin	BGC0000176.1	transAT-PKS	KKLW	CP054714.1	10	2,260,571	2,366,747	difficidin	Polyketide + NRP	100%
Subtilosin	BGC0000602.1	Sactipeptide	KKD1	CP054584.1	9	3,840,443	3,862,055	subtilosin	RiPP:Thiopeptide	100%
Plantazolicin	BGC0000569.1	LAP	KKLW	CP054714.1	2	717,313	740,49	plantazolicin	RiPP:LAP	100%
Amylocyclicin	BGC0000616.1	circular bacteriocin	KKLW	CP054714.1	12	3,048,069	3,048,404	amylocyclicin	RiPP-like	100%
		NRPS	KKLW	CP054714.1	11	2,849,731	2,904,624			
		T3PKS	KKD1	CP054584.1	7	2,349,606	2,390,703			
		T3PKS	KKLW	CP054714.1	9	2,103,065	2,144,165			
		Lanthipeptide-class III	KKD1	CP054584.1	2	852,287	874,884			
		Terpene	KKD1	CP054584.1	3	1,151,502	1,171,944			
		Terpene	KKLW	CP054714.1	4	1,064,513	1,081,742			

**Terpene**	**KKD1**	**CP054584.1**	**6**	2,147,595	2,169,493
Terpene	KKLW	**CP054714.1**	8	2,014,696	2,036,579
